# Anti-PTK7 Monoclonal Antibodies Suppresses Oncogenic Phenotypes in Cellular and Xenograft Models of Triple-Negative Breast Cancer

**DOI:** 10.3390/cells14030181

**Published:** 2025-01-24

**Authors:** Min Ho Kim, Mi Kyung Park, Han Na Park, Seung Min Ham, Ho Lee, Seung-Taek Lee

**Affiliations:** 1 Department of Biochemistry, College of Life Science and Biotechnology, Yonsei University, Seoul 03722, Republic of Korea; tomorrow0918@naver.com (M.H.K.); qkrgkssk1024@naver.com (H.N.P.); mongsil1115@naver.com (S.M.H.); 2 Department of Cancer Biomedical Science, Graduate School of Cancer Science and Policy, National Cancer Center, Goyang 10408, Republic of Korea; mkpark@hsmu.ac.kr; 3 Department of Biomedical Science, Hwasung Medi-Science University, Hwaseong 18274, Republic of Korea

**Keywords:** MDA-MB-231 cells, monoclonal antibody, oncogene, PTK7, receptor protein tyrosine kinase, triple-negative breast cancer

## Abstract

Protein tyrosine kinase 7 (PTK7), a catalytically defective receptor protein tyrosine kinase, is frequently upregulated in various cancers, including triple-negative breast cancer (TNBC), and is associated with poor clinical outcomes. Analysis of The Cancer Genome Atlas (TCGA) data confirmed that PTK7 mRNA expression is significantly higher in TNBC tumor tissues compared with adjacent normal tissues and non-TNBC breast cancer subtypes. Kaplan–Meier survival analysis demonstrated a strong correlation between high PTK7 expression and worse relapse-free survival in TNBC patients (HR = 1.46, *p* = 0.015). In vitro, anti-PTK7 monoclonal antibodies (mAbs) significantly reduced proliferation, wound healing, migration, and invasion in TNBC MDA-MB-231 cells. Ki-67 immunofluorescence assays revealed substantial decreases in cell proliferation following treatment with PTK7 mAbs (32-m, 43-m, 50-m, and 52-m). Moreover, actin polymerization, a critical process in cell migration and invasion, was markedly impaired upon PTK7 mAb treatment. In vivo, PTK7 mAbs significantly reduced tumor volume and weight in a TNBC xenograft mouse model compared with controls. Treated tumors exhibited decreased expression of Ki-67 and vimentin, indicating reduced proliferation and epithelial-to-mesenchymal transition. These findings highlight PTK7 as a promising therapeutic target in TNBC and demonstrate the potent anti-cancer effects of PTK7-neutralizing mAbs both in vitro and in vivo. Further exploration of PTK7-targeted therapies, including humanized mAbs and antibody-drug conjugates, is warranted to advance treatment strategies for PTK7-positive TNBC.

## 1. Introduction

Breast cancer (BC) is the most common cancer among women and a major cause of morbidity and mortality worldwide [1,2]. In the United States, in 2024, BC accounted for 32% of all cancers diagnosed in women and was the leading cause of cancer-related mortality among women aged 20 to 49 years [3]. BC is a heterogeneous disease, exhibiting significant inter- and intratumoral variability [4,5,6]. It can be classified into three major subtypes based on receptor status: (1) hormone receptor-positive (HR+) BC, characterized by the presence of estrogen receptor (ER) and progesterone receptor (PR), (2) HER2-positive (HER2+), involving HER2 overexpression, and (3) triple-negative BC (TNBC), which lacks ER, PR, and HER2 expression [7].

HR+ BCs are commonly treated with hormone therapies such as tamoxifen (an ER blocker) and aromatase inhibitors like anastrozole, letrozole, and exemestane, which inhibit estrogen production [8,9]. HER2+ BCs are treated with HER2-targeted therapies, such as trastuzumab, often combined with chemotherapy, like docetaxel, or antibody-drug conjugates (ADCs) like trastuzumab emtansine [10,11]. However, TNBC lacks a suitable therapeutic target and is primarily managed with traditional chemotherapy [12,13]. TNBCs with BRCA mutations may respond to PARP inhibitors, such as olaparib or talazoparib, and some TNBCs exhibit responsiveness to immune checkpoint inhibitors, like pembrolizumab or atezolizumab [14,15,16,17].

Protein tyrosine kinase 7 (PTK7), also known as colon carcinoma kinase 4 (CCK4), is a catalytically defective receptor protein tyrosine kinase. Despite lacking catalytic activity, PTK7 plays pivotal roles in development, particularly as a regulator of planar cell polarity (PCP), critical for neural tube closure, axon guidance, and epithelial organization. PTK7 interacts with PCP pathway components like Vangl2, Dishevelled, and Frizzled [18,19,20] and binds non-canonical Wnt ligands (e.g., Wnt5a and Wnt11) to regulate cell polarity and migration [21,22]. PTK7 also interacts with canonical Wnt signaling components, such as β-catenin, though evidence suggests PTK7 may inhibit canonical Wnt signaling rather than activate it [22,23,24,25].

PTK7 is upregulated in various cancers, including BC, colon, gastric, lung, esophageal, ovarian cancers, and acute myeloid leukemia, where it regulates cell adhesion, migration, proliferation, and survival [26]. It is implicated in tumorigenesis, angiogenesis, metastasis, and poor prognosis [27].

Signaling pathways, such as Wnt/β-catenin activation, epithelial-to-mesenchymal transition (EMT) pathways, and apoptosis suppression, contribute to PTK7’s oncogenic roles [23,27]. PTK also activates RPTK signaling, such as kinase insert domain receptor (KDR) activation in vascular endothelial growth factor (VEGF)-induced angiogenesis [28,29]. Additionally, PTK7 promotes proliferation, migration, and invasiveness of esophageal squamous cell carcinoma (ESCC) cells by activating fibroblast growth factor receptor 1 (FGFR1) [30,31,32] and contributes to epidermal growth factor receptor (EGFR)/Akt signaling in TNBC cells [33]. PTK7 knockdown reduces growth-factor-induced phosphorylation of FGFR1 and EGFR in MDA-MB-231 cells, indicating its role in RPTK activation [34].

PTK7 is frequently overexpressed in BC and correlates with poor clinical outcomes, including increased tumor size, lymph node metastasis, and reduced survival [35]. It is also highly expressed in tumor-initiating cells derived from TNBC, ovarian, and non-small-cell lung cancers [6]. PTK7-negative tumors exhibit improved disease-free survival, particularly with anthracycline treatment, suggesting its role in chemotherapy resistance [36]. PTK7 knockdown consistently reduces tumorigenic effects in BC [33,34].

Recent studies demonstrate that PTK7-neutralizing monoclonal antibodies (mAbs) inhibit VEGF-induced angiogenesis, block KDR activation, and decrease proliferation and migration in ESCC models [29]. This study aims to evaluate the anti-cancer activity of four PTK7 mAbs (32-m, 43-m, 50-m, and 52-m) in TNBC MDA-MB-231 cells, assessing their effects on oncogenic phenotypes and tumorigenesis in mouse xenograft models. These findings could provide insights into PTK7 mAbs as potential therapeutic agents for TNBC and other PTK7-positive cancers.

## 2. Materials and Methods

### 2.1. Analysis of PTK7 Expression in Breast Cancer Patients

PTK7 expression levels in breast cancer tissues and adjacent normal tissues were analyzed using RNA-sequencing (RNA-seq) data from The Cancer Genome Atlas (TCGA), accessed through the Breast Cancer Integrative Platform (BCIP; http://www.omicsnet.org/bcancer/, accessed on 6 January 2024). To assess the prognostic significance of PTK7 in breast cancer, the Kaplan–Meier Plotter mRNA breast cancer database (http://kmplot.com/analysis/, accessed on 6 January 2024) was utilized with probe ID 207011_s_at. The processed and normalized data were obtained directly from the respective platforms without any additional transformations.

### 2.2. Cell Culture

Human TNBC MDA-MB-231 cells were obtained from the Korean Cell Line Bank (Seoul, Republic of Korea) and cultured in Dulbecco’s Modified Eagle Medium (DMEM; Gibco of Thermo Fisher Scientific, Grand Island, NY, USA) supplemented with 5% FBS (Gibco), 2.5 mM sodium glutamine, 100 U/mL penicillin, and 100 μg/mL streptomycin. Cells were maintained in a humidified atmosphere of 5% CO_2_ and 95% air at 37 °C.

### 2.3. Anti-PTK7 mAbs

The production and purification of anti-PTK7 mAbs (32-m, 43-m, 50-m, and 52-m) were carried out as previously described [29].

### 2.4. Cell Proliferation Assay

Subconfluent MDA-MB-231 cells were detached and resuspended in fresh medium. Cells (4 × 10^3^/well) were seeded on confocal dishes (Corning, Bedford, MA, USA) and incubated with 3 mL DMEM supplemented with 5% FBS, with or without PTK7 mAbs (10 μg/mL), for 24 h. After incubation, cells were fixed with 3.7% paraformaldehyde (PFA) for 15 min, permeabilized with 0.2% Triton-X 100 for 10 min, and blocked with 3% bovine serum albumin (BSA) for 30 min. Immunostaining was performed overnight at 4 °C using 2.5 μg/mL of mouse anti-Ki-67 antibody (#9449; Cell Signaling Technology, Danvers, MA, USA). After washing with phosphate-buffered saline (PBS), cells were incubated for 2 h with 1.0 U/mL of Rhodamine Red-X-conjugated anti-mouse IgG antibody (Thermo Fisher Scientific, Grand Island, NY, USA) to visualize proliferating cells. Nuclear staining was performed using Hoechst 33258 (2.0 μg/mL). Images were obtained using a confocal fluorescence microscope (LSM880; Carl Zeiss, Feldbach, Switzerland).

### 2.5. Wound Healing Assay

MDA-MB-231 cells grown in 12-well plates were starved in serum-free DMEM for 24 h. Wounds were introduced by scraping the monolayer with 1000 μL micropipette tips, and cells were incubated in DMEM containing 5% FBS with or without PTK7 mAbs (10 μg/mL) for 24 h. Images of the wounds were captured using light microscopy before treatment and after 24 h of incubation. Images were captured using an optical microscope with 100× objective lens.

### 2.6. Chemotactic Migration and Invasion Assays

For the migration assay, MDA-MB-231 cells were starved in serum-free DMEM for 24 h. Cells in serum-free DMEM (1 × 10^5^ cells/0.1 mL) were preincubated with or without PTK7 mAbs (10 μg/mL) for 30 min, then loaded into the upper chamber of Transwell inserts (8 μm pore size, Corning), of which the lower surface was coated with 10 μL of 0.1% gelatin and dried for 30 min at room temperature. The lower chambers were filled with 0.65 mL DMEM supplemented with 5% FBS as a chemoattractant. After incubation for 24 h at 37 °C, migrated cells were fixed with 3.7% PFA, stained with 0.2% crystal violet, and solubilized in 1% sodium dodecyl sulfate. Absorbance was measured at 600 nm. For the invasion assay, the upper surfaces of Transwell inserts were coated with 25 μg Matrigel (Corning) and dried at 37 °C for 60 min. Cells in serum-free DMEM (7 × 10^4^ cells/0.1 mL) were preincubated with or without PTK7 mAbs (10 μg/mL) for 30 min, then loaded into the upper chambers. The lower chambers were filled with 0.65 mL serum-free DMEM and DMEM supplemented with 5% FBS as a chemoattractant. After incubation for 48 h at 37 °C, invaded cells were processed as described above. Images of migrated and invaded cells were captured using an optical microscope with 40× objective lens.

### 2.7. Analysis of Actin Filament Distribution in Cells

MDA-MB-231 cells were plated on confocal dishes and incubated in 5% FBS DMEM with or without PTK7 mAbs (10 μg/mL) for 24 h. After washing with PBS, cells were fixed with 3.7% PFA for 15 min, stained with 1 μg/mL of FITC-phalloidin (Thermo Fisher Scientific, Waltham, MA, USA) for 1 h at 25 °C, and counterstained with Hoechst 33258 (2.0 μg/mL). Images were captured using a confocal microscope with 200× objective lens.

### 2.8. Analysis of Tumor Growth in Xenograft Mouse Model of TNBC

BALB/c-nu/nu mice (4–5 weeks old, male) were obtained from Orient Bio, Inc. (Gyeonggi, Republic of Korea). MDA-MB-231 cells (1 × 10^6^) resuspended in 200 µL of a 1:1 mixture of Matrigel and PBS were injected subcutaneously into the dorsal regions of mice. Once tumor growth was established, PTK7 mAbs (10 mg/kg) or PBS (control) were administered intravenously twice a week for 3 weeks. Tumor volumes were measured twice weekly using calipers (length/2 × width × width). Two weeks after completing antibody treatment, mice were sacrificed, and tumors were excised to measure tumor weight. Tumors were then fixed in 4% PFA and processed for histological and immunohistochemical (IHC) analyses.

### 2.9. IHC Staining

Paraffin-embedded tumor sections (4 μm) were deparaffinized and subjected to antigen retrieval in sodium citrate buffer (10 mM sodium citrate and 0.05% Tween 20, pH 6.0) for 10 min on a hot plate. Sections were blocked, incubated overnight with the primary antibodies, and then with horseradish peroxidase-conjugated secondary antibody (Invitrogen, Carlsbad, CA, USA) for 1 h at 25 °C. Staining was performed using 3,3-diaminobenzidine (Thermo Fisher Scientific) for 5 min at 25 °C, and slides were counterstained with hematoxylin. Quantitative analysis was conducted using Vectra^®^ Polaris^TM^ Imaging System and inForm software (version 2.6) (Akoya Biosciences, Waltham, MA, USA).

### 2.10. Ethics Statement

All animal studies were approved by the Institutional Animal Care and Use Committee of the National Cancer Center Research Institute (NCC-20-517 and NCC-24-1022, which is an Association for Assessment and Accreditation of Laboratory Animal Care International accredited facility that adheres to the Institute of Laboratory Animal Resources guidelines.

### 2.11. Statistical Analysis

Data from at least three independent experiments were expressed as mean ± standard deviation. Unless otherwise specified, statistical significance was determined using Student’s *t*-test performed in Excel or GraphPad Prism 9 software. A *p*-value of less than 0.05 was considered statistically significant.

## 3. Results

### 3.1. PTK7 Is Upregulated in TNBC and Inversely Correlates with Relapse-Free Survival in TNBC

**Figure 1:**
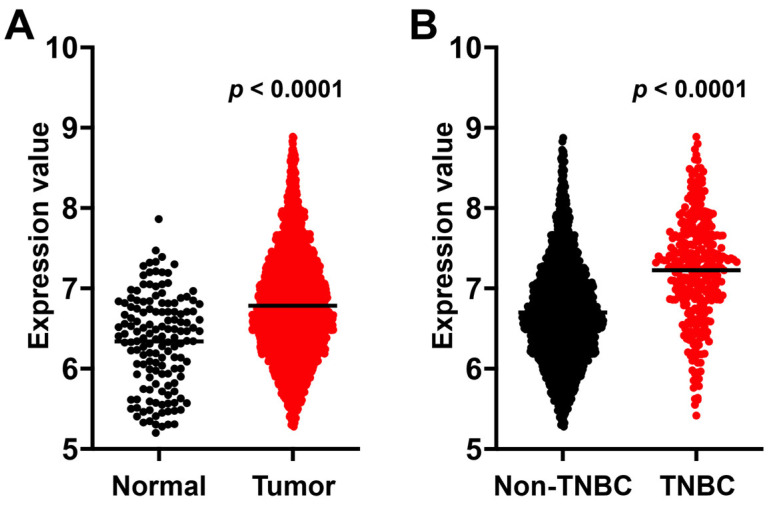
PTK7 mRNA expression in BC tumor and adjacent normal tissues and in non-TNBC and TNBC tumor tissues. Expression levels of PTK7 mRNA in BC and adjacent normal tissues (**A**) and in non-TNBC and TNBC tissues (**B**) were analyzed using TCGA data through the Breast Cancer Integrative Platform (http://www.omicsnet.org/bcancer/ accessed on 6 January 2024). Bars represent the median expression levels in each group. Statistical analysis was performed using the Mann–Whitney U test.

Using data from TCGA, we analyzed PTK7 mRNA expression in both tumor and adjacent normal tissues, as well as in non-TNBC and TNBC. The results show that PTK7 mRNA levels were significantly higher in BC tumor tissues compared with adjacent normal tissues. Furthermore, tumor tissues from TNBC exhibited significantly higher PTK7 mRNA expression than non-TNBC tumor tissues (Figure 1A,B).

**Figure 2:**
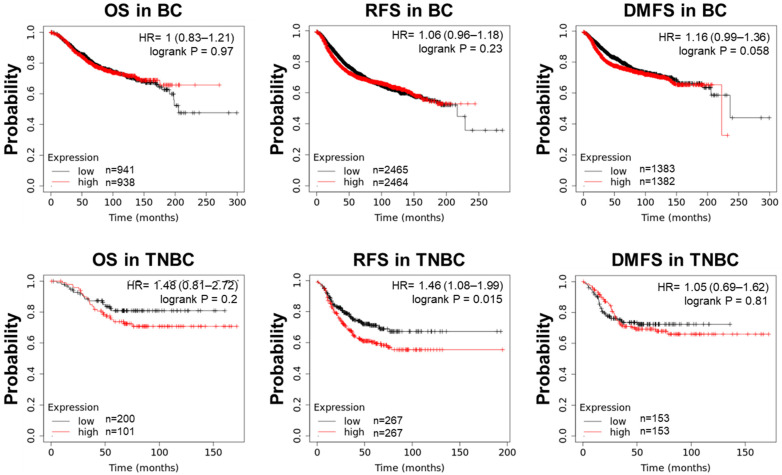
Survival analysis based on PTK7 mRNA expression in BC and TNBC cohorts. The prognostic significance of PTK7 mRNA expression in BC and TNBC was evaluated using the Kaplan–Meier Plot database (http://kmplot.com/analysis/ accessed on 6 January 2024). OS and RFS were analyzed in all BC patients, while DMFS was assessed in patients with lymph node-positive cancer. The number of patients with low and high PTK7 expression used in each analysis is indicated. Statistical significance was determined using log-rank tests. A hazard ratio (HR) below 1 indicates better survival probabilities for the low-PTK7-expression group compared with the high-PTK7-expression group.

The prognostic impact of PTK7 mRNA expression on overall survival (OS), relapse-free survival (RFS), and distant metastasis-free survival (DMFS) in BC and TNBC patients was assessed using Kaplan–Meier analysis. In TNBC patients, PTK7 expression was significantly associated with poorer RFS, with a hazard ratio of 1.46 (1.08–1.99) and a log-rank *p*-value of 0.015, indicating worse survival outcomes with high PTK7 expression (Figure 2). While no significant association was found between PTK7 expression and OS, RFS, or DMFS in BC patients, or between PTK7 expression and OS or DMFS in TNBC patients, hazard ratios above 1.00 for RFS and DMFS in BC patients, as well as for OS and DMFS in TNBC patients suggest a potential negative effect of high PTK7 expression on survival. These findings underscore the prognostic relevance of PTK7 in TNBC, particularly in predicting relapse-free survival.

### 3.2. Anti-PTK7 mAbs Reduces Proliferation of MDA-MB-231 TNBC Cells

**Figure 3:**
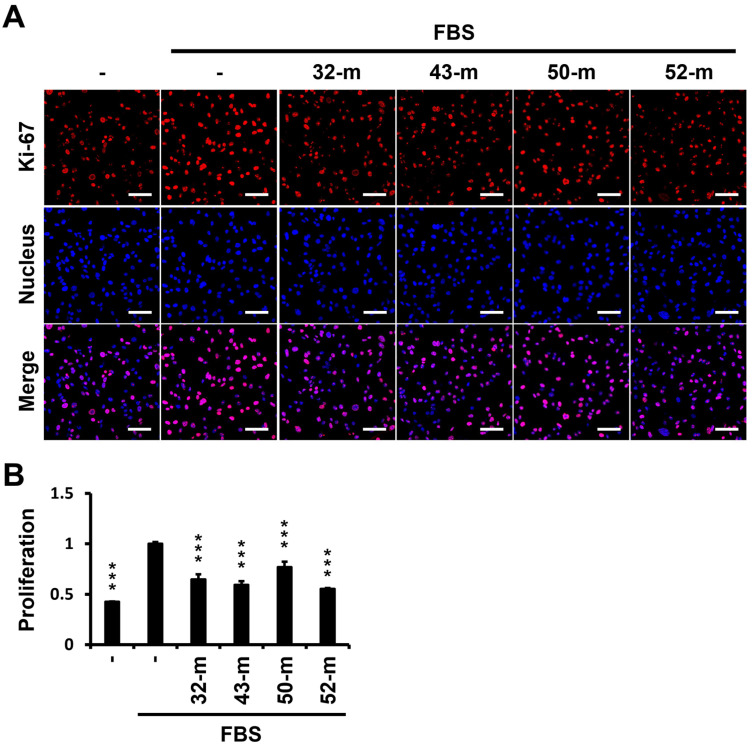
Effect of anti-PTK7 mAbs on proliferation in MDA-MB-231 cells. MDA-MB-231 cells were incubated in DMEM without or with 5% FBS and treated with anti-PTK7 mAbs (32-m, 43-m, 50-m, and 52-m; 10 μg/mL) for 24 h. Proliferating cells were detected by staining with anti-Ki-67 and Rhodamine Red-X-conjugated anti-mouse IgG antibodies, with nuclear staining performed with Hoechst 33258. (**A**) Representative images were captured using confocal fluorescence microscopy at 200× magnification. (**B**) Quantification of Ki-67 staining was performed using ImageJ software (version 1.53), normalized to nuclear staining. Each value represents the mean ± SD of three independent experiments, with statistical significance denoted as *** *p* < 0.001 compared with the FBS-treated control group. The scale bar represents 100 μm.

PTK7 plays a key role in cell proliferation, with previous studies showing that its knockdown hampers TNBC tumorigenic phenotypes. Here, we evaluated the impact of four anti-PTK7 mAbs (32-m, 43-m, 50-m, and 52-m) on the proliferation of MDA-MB-231 cells using Ki-67 as a proliferation marker. Under FBS-depleted conditions, proliferation, measured as the relative Ki-67 level normalized to nuclear staining, was reduced to 42.7 ± 2.0% relative to the FBS-treated control group. Treatment with anti-PTK7 mAbs (32-m, 43-m, 50-m, and 52-m) significantly reduced Ki-67 expression: 64.9 ± 3.8% for 32-m, 59.3 ± 2.2% for 43-m, 77.1 ± 3.0% for 50-m, and 55.5 ± 4.2% for 52-m compared with the FBS-treated control (Figure 3). These findings indicate that anti-PTK7 mAbs significantly reduce cell proliferation, with effectiveness ranked as 52-m, 43-m, 32-m, and 50-m.

### 3.3. Anti-PTK7 mAbs Inhibit Oncogenic Behaviors in MDA-MB-231 Cells

**Figure 4:**
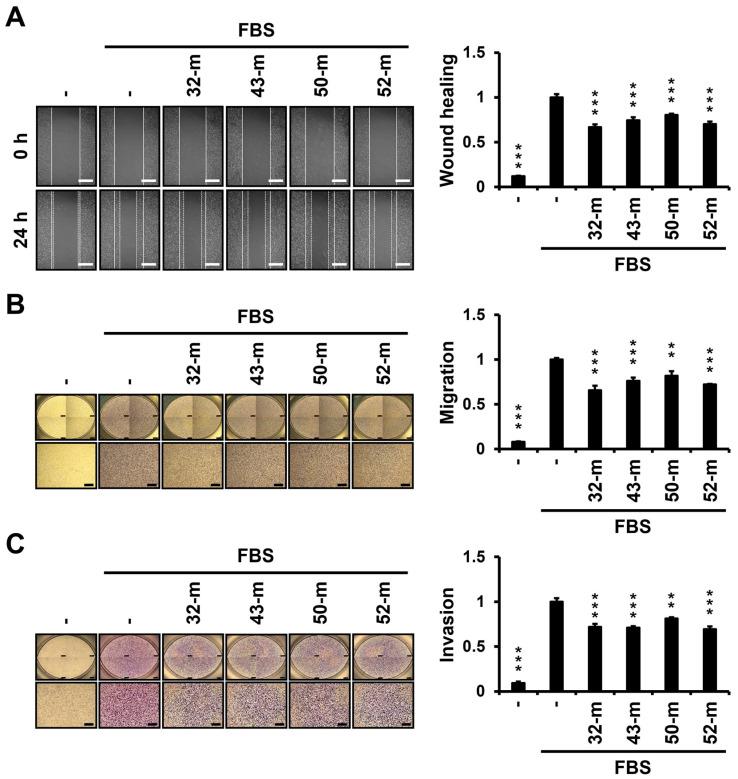
Effect of anti-PTK7 mAbs on wound healing, migration, and invasion in MDA-MB-231 cells. (**A**) Wound healing was evaluated 24 h post-scratch in monolayer culture after treatment with anti-PTK7 mAbs. Representative micrographs (100× magnification) were captured at 0 h and 24 h post-antibody application. The wound-healing area was quantified using ImageJ software. The graph shows the relative wound area in anti-PTK7 mAb-treated groups normalized to the FBS-treated control group. The scale bar represents 1 mm. (**B**) Chemotactic migration was measured 24 h post-antibody treatment using a Transwell apparatus. (**C**) Chemotactic invasion was measured 48 h post-antibody treatment using a Transwell apparatus. Migrated and invaded cells on the bottom of the Transwell were stained with crystal violet for visualization. Representative micrographs (40× magnification) were captured at 24 h for migration and 48 h for invasion. Stained cells were solubilized in 1% sodium dodecyl sulfate, and absorbance was measured at 600 nm. Graphs show the relative absorbance of the anti-PTK7 mAbs-treated groups, normalized to the FBS-treated control group. The scale bar represents 500 μm. Each value represents the mean ± SD of three independent experiments: ** *p* < 0.01 or *** *p* < 0.001 vs. FBS-treated control.

To assess the impact of anti-PTK7 mAbs on oncogenic behaviors beyond proliferation, we conducted wound healing, migration, and invasion assays in MDA-MB-231 cells following treatment with PTK7 mAbs. In wound healing assays, the FBS-depleted group showed a reduction to 11.9 ± 0.6% compared with the FBS-treated control. Treatment with anti-PTK7 mAbs significantly decreased wound healing rates to 66.6 ± 3.3% for 32-m, 74.2 ± 3.5% for 43-m, 80.3 ± 1.3% for 50-m, and 70.2 ± 2.9% for 52-m (Figure 4A). In chemotactic migration assays, the FBS-depleted group was reduced to 8.1 ± 0.4% relative to the FBS-treated control, while anti-PTK7 mAbs reduced migration to 65.5 ± 5.1% for 32-m, 76.2 ± 3.8% for 43-m, 81.7 ± 5.2% for 50-m, and 72.0 ± 0.7% for 52-m (Figure 4B). In chemotactic invasion assays, the FBS-depleted group showed a reduction to 9.2 ± 1.6% compared with the FBS-treated control, with anti-PTK7 mAbs reducing invasion to 71.9 ± 3.6% for 32-m, 71.1 ± 1.8% for 43-m, 81.4 ± 1.4% for 50-m, and 69.5 ± 3.0% for 52-m (Figure 4C). These findings confirm the anti-tumor effects of anti-PTK7 mAbs on MDA-MB-231 cells at the cellular level, with relative efficacy ranked as 32-m, 52-m, 43-m, and 50-m.

### 3.4. Anti-PTK7 mAbs Reduce Actin Polymerization in MDA-MB-231 Cells

**Figure 5:**
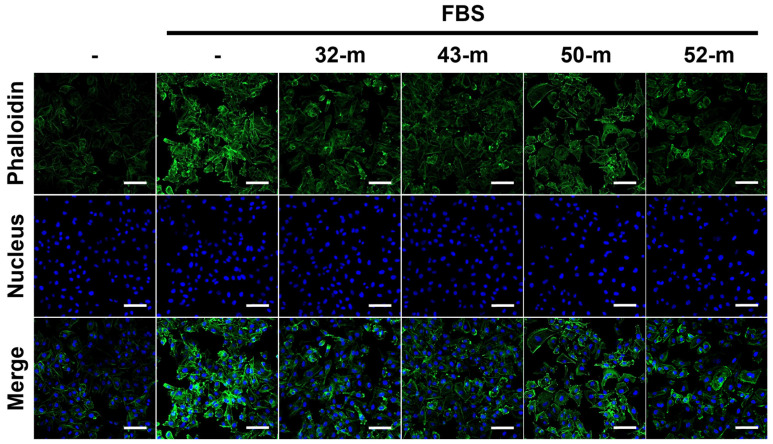
Effect of anti-PTK7 mAbs on actin polymerization in MDA-MB-231 cells. MDA-MB-231 cells were cultured in DMEM supplemented with or without 5% FBS and treated with anti-PTK7 mAbs (10 μg/mL) for 24 h. Actin filaments visualized using FITC-phalloidin are shown alongside nuclei staining (Hoechst 33258). Images were captured via confocal fluorescence microscopy at 200× magnification. The scale bar represents 100 μm.

Actin polymerization, essential for migration and invasion, in MDA-MB-231 cells treated with or without PTK7 mAbs was evaluated using immunofluorescence staining with fluorescein isothiocyanate (FITC)-conjugated phalloidin. In the FBS-treated control group, strong actin filament polymerization was observed in the cortical regions, particularly at leading edges and focal contacts. In contrast, treatment with anti-PTK7 mAbs (32-m, 43-m, 50-m, and 52-m) significantly reduced actin polymerization to levels close to those in FBS-depleted conditions (Figure 5). These findings suggest that anti-PTK7 mAb treatment impairs the migratory and invasive capabilities of TNBC cells, at least in part, by effectively reducing actin polymerization.

### 3.5. Anti-PTK7 mAbs Reduce Tumor Volume and Weight in TNBC Xenograft Mouse Model

**Figure 6:**
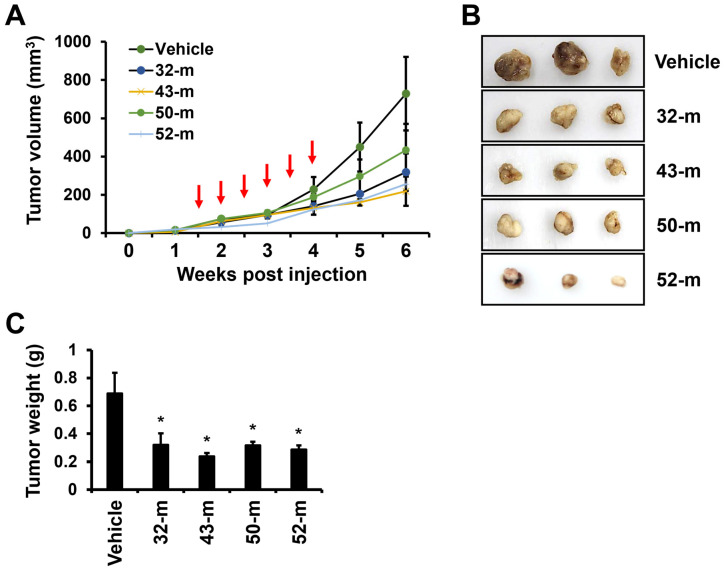
Inhibition of tumorigenesis by anti-PTK7 mAbs in the TNBC xenograft model. The TNBC xenograft mouse model was established by subcutaneous injection of MDA-MB-231 cells into the dorsal regions of nude mice. PTK7 mAbs (10 mg/kg) were administered intravenously via tail veins twice a week for three weeks (indicated by red vertical arrows). Mice were euthanized 2 weeks after the final treatment, and tumors were collected for analysis. (**A**) Tumor volume was monitored throughout the in vivo experiment. (**B**) Images of tumors excised from euthanized mice are shown. (**C**) Tumor weight data are presented graphically. Each value represents the mean ± SD of three mice: * *p* < 0.05 vs. the vehicle control group.

To evaluate the in vivo anti-cancer effects of anti-PTK7 mAbs on TNBC, we utilized the tumorigenic potential of MDA-MB-231 cells in a xenograft mouse model. Mice were subcutaneously injected with MDA-MB-231 cells to induce tumor formation, and anti-PTK7 mAbs (10 mg/kg) were administered intravenously twice a week for 3 weeks. Two weeks after the final treatment, the mice were euthanized, and the tumors were collected for analysis. Tumor volumes in the vehicle group were 728.7 mm^3^, while significant reductions were observed in the treatment groups: 43.7 ± 10.9% for 32-m, 30.0 ± 8.5% for 43-m, 59.4 ± 15.3% for 50-m, and 35.1 ± 25.9% for 52-m, compared with the vehicle control group (Figure 6A). Similarly, tumor weights in the vehicle control group averaged 0.69 g, with significant reductions observed in the treatment groups: 46.5 ± 12.0% with 32-m, 34.4 ± 3.6% with 43-m, 46.0 ± 3.6% with 50-m, and 41.6 ± 4.1% with 52-m, compared with the vehicle control group (Figure 6B). These results demonstrate that anti-PTK7 mAbs significantly reduce both tumor volume and weight in the TNBC xenograft mouse model, with relative efficacy ranked as 43-m, 52-m, 32-m, and 50-m. Among the treatment groups, 43-m and 52-m exhibited significantly lower tumor weights compared with 32-m and 50-m.

**Figure 7:**
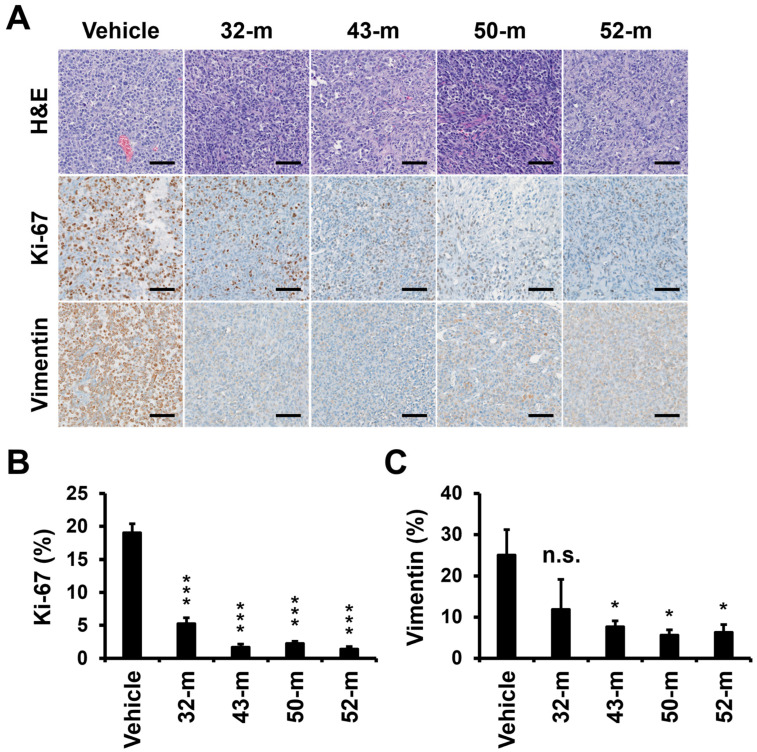
H&E and IHC staining of Ki-67 and vimentin in xenograft tumor sections. (**A**) Representative images of H&E and IHC staining for Ki-67 and vimentin are shown. Ki-67 (**B**) and vimentin (**C**) levels in IHC images were quantitated using ImageJ software. Each value represents the mean ± SD of three mice. * *p* < 0.05, *** *p* < 0.001 vs. vehicle control group; n.s.: not significant. The scale bar represents 100 μm.

To further investigate the effects of anti-PTK7 mAbs on tumor characteristics, hematoxylin and eosin (H&E) and IHC staining were performed on tumor sections to evaluate Ki-67 (proliferation marker) and vimentin (mesenchymal marker). Treatment with PTK7 mAbs significantly reduced Ki-67 levels to 5.27 ± 0.90% for 32-m, 1.71 ± 0.44% for 43-m, 2.24 ± 0.37% for 50-m, and 1.39 ± 0.40% for 52-m, compared with 19.0 ± 1.4% in the vehicle group. Similarly, vimentin levels decreased to 11.9 ± 7.3% for 32-m, 7.67 ± 1.4% for 43-m, 5.60 ± 1.3% for 50-m, and 6.27 ± 1.9% for 52-m, compared with 25.0 ± 6.2% in the vehicle group (Figure 7). These results confirm that anti-PTK7 mAbs reduce proliferation and mesenchymal marker expression, with the relative efficacy ranked as 52-m, 43-m, 50-m, and 32-m. Statistically significant differences were not observed between the PTK7 mAb-treated groups, except that 32-m showed a significantly lower Ki-67 level compared with the other mAbs.

## 4. Discussion

PTK7, a catalytically defective RPTK, lacks enzymatic activity due to alteration in its tyrosine kinase domain [37,38,39]. This structural feature makes the development of small-molecule inhibitors targeting PTK7 challenging. To overcome this obstacle, PTK7-neutralizing antibodies have been developed to inhibit its carcinogenic functions [40]. These antibodies effectively block oncogenic phenotypes in vitro, suppress tumorigenesis in xenograft models, such as in ESCC [40], and inhibit VEGF-induced angiogenesis both ex vivo and in vivo [29].

We and others have shown that PTK7 is frequently overexpressed in BC, particularly in TNBC, compared with adjacent normal tissues [6,33,34,41]. Analysis of the TCGA database further supports these findings. The overexpression of PTK7 in breast cancer suggests potential applications in early cancer diagnosis, especially through the use of PTK7-specific aptamers [42,43]. However, the prognostic significance of PTK7 expression has been inconsistent. Ataseven et al. [36] reported no significant difference in DFS or OS based on PTK7 expression, though patients with PTK7-negative tumors demonstrated better DFS, especially when treated with anthracycline-based therapies. Cui et al. [33] observed no significant differences in survival between PTK7-high and PTK7-low patients across luminal A, luminal B, or HER2-positive BC subtypes but found significantly worse RFS in TNBC patients with high PTK7 expression. Similarly, Lacey et al. [41] reported that PTK7 mRNA expression correlated with survival outcomes in BC patients, particularly in patients with moderate to poor prognosis. Our study corroborates these findings, showing that high PTK7 expression is significantly associated with worse RFS in TNBC (*p* = 0.015). Although statistical significance was not observed for other measures, hazard ratios suggested a trend toward poor outcomes in high PTK7-expressing BC and TNBC. These results highlight the potential prognostic value of PTK7, particularly in TNBC.

TNBC, defined by the absence of ER, PR, and HER2, has limited treatment options. In this study, we investigated the potential of PTK7-neutralizing mAbs in the treatment of TNBC. Although MDA-MB-231 cells exhibit low PTK7 expression [6,34,44,45], their reliability for engraftment in nude mice made them a suitable choice for xenograft studies. Moreover, PTK7 knockdown in these cells significantly reduces tumorigenic functions such as proliferation, adhesion, migration, and invasion [34]. Therefore, despite their low PTK7 expression, MDA-MB-231 cells were used to assess the anticancer activity of PTK7 mAbs at both cellular and in vivo levels.

We demonstrated that four PTK7 mAbs (32-m, 43-m, 50-m, and 52-m) significantly suppressed tumorigenic functions in MDA-MB-231 cells. Ki-67 staining revealed a marked reduction in proliferation. Similarly, the four PTK7 mAbs markedly reduced wound healing, chemotactic migration, and invasion. These findings underscore the potential of PTK7 mAbs to target tumor progression in TNBC.

Actin filament polymerization drives cell motility, an essential process in cancer migration and invasion [46]. Reduced actin polymerization has been linked to impaired migration and invasion [47,48,49]. Consistent with findings in ESCC cells [40], we observed that PTK7 mAbs significantly inhibited actin polymerization in MDA-MB-231 cells, suggesting that their anti-migratory and anti-invasive effects may be mediated by disrupting cytoskeletal dynamics.

It was previously reported that xenograft tumor growth was significantly reduced in mice when TNBC cells with PTK7 knockdown were used, compared with mice xenografted with TNBC cells without PTK7 knockdown [33]. Similarly, we observed that PTK7 mAbs exhibited comparable anti-cancer effects to PTK7 knockdown. Specifically, treatment with PTK7 mAbs 32-m and 43-m in an ESCC xenograft mouse model using KYSE-30 cells showed notable anti-cancer effects [40]. Additionally, PTK7 mAbs (32-m, 43-m, 50-m, and 52-m) inhibited VEGF-induced angiogenesis in HUVECs and mice [29]. Based on these findings, we assessed the anti-cancer effects of PTK7 mAbs (32-m, 43-m, 50-m, and 52-m) in a TNBC xenograft mouse model using MDA-MB-231 cells. The results revealed significant reductions in tumor weight and volume in the PTK7 mAb-treated groups compared with controls, with the efficacy as 43-m, 52-m, 32-m, and 50-m. Furthermore, IHC analysis demonstrated that PTK7 mAb treatment significantly decreased Ki-67 expression, a proliferation marker, and vimentin expression, a mesenchymal cell marker, in xenograft tumors. These findings suggest that PTK7 mAbs inhibit tumor growth by targeting both proliferation and EMT in vivo. As neutralizing antibodies, PTK7 mAbs specifically target PTK7-positive cells, underscoring their potential as a therapeutic agent for PTK7-positive cancers.

Antibodies that bind to selected antigens on the cell surface initiate internalization of the entire surface complex, allowing for intracellular delivery [50]. Antibodies with high internalization efficiency are being developed as ADCs with potent cytotoxic payloads. For example, cofetuzumab pelidotin (PF-06647020), a PTK7 mAb conjugated to a microtubule inhibitor via a cleavable linker, has demonstrated strong anti-tumor activity. Targeting PTK7 with ADCs has shown considerable promise, particularly in reducing tumor-initiating cells and inducing tumor regression, including patient-derived xenografts [6,51]. Clinical trials further confirmed the potential of PTK7-targeting ADCs, showing significant anti-tumor activity in patients with non-small cell lung cancer [51]. Furthermore, MTX-13, another PTK7-targeting ADC carrying the topoisomerase I inhibitor exatecan, demonstrated sustained tumor regressions across a broader range of PTK7-positive tumors, suggesting its expanded therapeutic potential [52].

The payload in ADCs is critical for selectively killing target cells after internalization. Commonly used payloads include microtubule inhibitors, DNA-damaging agents, and RNA polymerase inhibitors [53]. Platinum salts, such as cisplatin and carboplatin, have shown efficacy in patients with BRCA1/2 mutations or genomic instability signatures [54,55]. Thus, selecting patients based on biomarkers and genomic profiles can further enhance ADC therapeutic outcomes.

Our findings demonstrate that PTK7-neutralizing mAbs effectively inhibit tumor growth, migration, invasion, and EMT in TNBC models, establishing them as a promising therapeutic option. Moreover, their potential integration into ADC platforms enhances their versatility as treatments for refractory PTK7-positive cancers, including TNBC. Further studies focusing on the development and application of humanized PTK7 antibodies are warranted to explore their clinical feasibility and optimize their therapeutic efficacy potential. By combining PTK7-neutralizing antibodies with advanced ADC strategies, the treatment landscape for PTK7-positive cancers, including TNBC, would be significantly improved.

## 5. Conclusions

Our study demonstrates the significance of PTK7 as a biomarker and therapeutic target in TNBC. We demonstrated that PTK7 is significantly upregulated in TNBC compared with both adjacent normal tissues and non-TNBC subtypes. High PTK7 expression is associated with poorer relapse-free survival, indicating its prognostic relevance in TNBC patients. Furthermore, anti-PTK7 mAbs effectively reduced key oncogenic behaviors such as proliferation, migration, invasion, and actin polymerization in TNBC MDA-MB-231 cells. In a TNBC xenograft mouse model, these mAbs exhibited significant anti-tumor effects, reducing tumor volume, weight, and the expression of proliferation and EMT markers. Together, these findings suggest that PTK7 plays a critical role in TNBC progression, and targeting PTK7 with specific mAbs holds promising therapeutic potential. Further development and clinical exploration of anti-PTK7 therapies could provide a valuable treatment option for PTK7-positive malignancies, such as TNBC.

## Data Availability

Data are contained within the article.
